# Strontium ranelate incorporated 3D porous sulfonated PEEK simulating MC3T3-E1 cell differentiation

**DOI:** 10.1093/rb/rbaa043

**Published:** 2020-11-28

**Authors:** Yingxiao Sun, Xingdan Liu, Ji Tan, Dan Lv, Wengang Song, Rui Su, Ling Li, Xuanyong Liu, Liping Ouyang, Yun Liao

**Affiliations:** 1 Department of Pharmacy, Tongren Hospital, Shanghai Jiao Tong University School of Medicine, Shanghai 200336, China; 2 State Key Laboratory of High Performance Ceramics and Superfine Microstructure, Shanghai Institute of Ceramics, Chinese Academy of Sciences, Changning District, Shanghai 200050, China; 3 Graduate School of Beihua University, Beihua University, Fengman District Jilin 132013, China

**Keywords:** polyetheretherketone, sulfonation, strontium ranelate, osseointegration

## Abstract

Polyetheretherketone (PEEK) has been used as an implant material because it has similar mechanical properties to natural bone. However, inferior osseointegration and bioinertness hamper the clinical application of PEEK. In this study, the surfaces of sulfonated three-dimensional (3D) PEEK porous structures were loaded with different concentrations of strontium ranelate, a compound commonly used in the treatment or prevention of osteoporosis by promoting bone formation and inhibiting bone resorption. Field-emission scanning electron microscopy was used to characterize the topography of the structures, elemental carbon, oxygen and strontium contents were measured by X-ray photoelectron spectroscopy, and surface zeta potentials and water-contact angle were also measured. The results indicated that strontium ranelate was successfully loaded onto the 3D porous structures. *In vitro* cellular results showed that strontium ranelate-treated sulfonated PEEK (SP-SR) strengthened the adhesion of MC3T3-E1 cells. The activity of alkaline phosphatase, collagen secretion and extracellular matrix mineralization deposition of MC3T3-E1 cells were also improved on the surface of SP-SR. These results indicate that SP-SR could serve a new implant candidate for surgical treatment.

## Introduction

Bones are critical organs of the human body, whose biomechanical properties were depended on the skeleton development and remodeling. The specialized mesenchymal cells, osteoblasts, are crucial for the mineralization of the skeleton and bone mass maintenance; In addition, osteoclasts also play an important role in bone resorption. The faults of osteoblast and osteoclast function are a major cause of terrible bone density [1], as it is seen in osteoporosis (OP), which is characterized by the loss of bone mass and an increased risk of fractures [2, 3]. With an aging population in China, the morbidity rate of osteoporotic fracture has increased [4, 5], increasing the need for implant materials.

Polyetheretherketone (PEEK) is a high-performance semicrystalline thermoplastic polymer with a glass transition temperature of 143°C, a crystallinity of 30–35% and an elastic modulus ranging from 3 to 4 GPa [6]. It has been widely used in surgical fields such as spine implant, joint implants [7] and trauma implants owing to the superior biomechanical properties [8, 9]. To improve the bioactive properties of PEEK, Accelerated Neutral Atom Beam technology can enhance the bioactivity of PEEK [10]. Titanium with a macro–micro–nano roughness surface can increase the rate of device integration with the surrounding bone tissue compared to PEEK [11]. Mg coated on PEEK is also a benefit for its bioactivity [12]. It is worth noting that the surface of PEEK can be treated to prepare a special three-dimensional (3D) porous structure having good biocompatibility and osseointegration [13–15]. The use of drug-delivery layers on SP structures has attracted more attention. For the patient with OP, loading drug that improves the bone remodel on the 3D porous structure can be an optional way for the disease fate.

Traditional therapeutic drugs for OP include calcium, drugs that inhibit osteoclast function, such as bisphosphonates, and the teriparatide that promotes osteogenic function. However, long-term OP therapy would increase risk of vascular events, allergies and osteonecrosis [16–18]. Strontium ranelate (SR) is FDA-approved clinical drug for OP therapy [19, 20]. This SR was reported to modulate bone metabolism by facilitating osteoblast proliferation and inhibiting osteoclast differentiation [21–23]. The oral bioavailability of SR is only 27% when dosed as indicated [24]. Large doses are inevitably to improve the local drug concentration. However, large doses often result in serious side effects, including drug allergy, increased risk of cardiovascular disease in patients with long-term use [25, 26], venous thrombosis and drug reaction with eosinophilia and systemic symptoms [27]. Reducing the side effects of drugs and exert their effects based on its multiple mechanisms on bone repair is an urgent problem in the treatment of OP. Local SR delivery can achieve satisfied effects with lower body dose. Studies have shown SR-mesoporous bioactive glass can enhance new bone formation [28, 29].

Consequently, in this study, SR was loaded onto the 3D porous SP surface. Field-emission scanning electron microscopy (FE-SEM), X-ray photoelectron spectroscopy (XPS), surface zeta potentials and water-contact angle measurements were used to characterize the surface properties of samples. The release of SR was determined by ultraviolet-visible spectrophotometry. Cell adhesion, proliferation, activity of alkaline phosphatase (ALP), collagen secretion and extracellular matrix (ECM) mineralization deposition were evaluated by mouse embryo osteoblast precursor cell line (MC3T3-E1).

## Experiment

### Sample preparation

From two different medical-grade PEEKs, wafer samples (diameter, 10 mm; thickness, 1 mm) and rectangular parallelepiped samples (20, 10 and 1 mm) were prepared. The wafer samples were used in 24-well tissue culture plates, and the rectangular parallelepiped samples were used in surface zeta potential measurements. One side of each sample was polished to an extremely smooth surface and then sequentially washed with acetone, ethanol and ultra-pure water. Samples were stirred at room temperature in sulfuric acid (95–98%) to form porous structures. Then, the samples were immersed in deionized water for 5 min and subjected to hydrothermal treatment at 120°C for 4 h [14]. Then, the samples were sterilized though ultraviolet radiation. The SR with three different concentrations (0.01, 0.1 and 1.0 mg/ml) were loaded onto the formed 3D SP network (SP-SR). Wafer samples were immersed in 1 ml 100% water solution with different concentrations of SR in a 24-well plate on an orbital shaker at 60 r/min at 37.5°C until the complete evaporation of water [30].

### Sample characterization

Field-emission scanning electron microscopy (Magellan 400, FEI, USA) was used to characterize the topography of samples. Elemental composition was measured by XPS (Axis UltraDLD, Japan). Sample surface wettability was checked using contact angle measurement (Automatic Contact Angle Meter, Model SL200B, Solon, China).

### Zeta potentials

The zeta potential of the sample surface was tested by a Surpass electrokinetic analyzer （Anton Parr, Austria）.Specifically, the samples (1 cm × 2cm × 1mm) were loaded in parallel on the sample stages with a gap of 100 ± 5μm. The 0.001M KCl solution was used as electrolyte. Then the pH varied from 10 to 3 adjusted by the 0.05M HCl solution, and hence the potential of the sample surface can be obtained according to the Helmholtz–Smoluchowski equation:

$$\zeta =\frac{dU}{dP}\times \frac{\eta }{\varepsilon \times \varepsilon_{0}}\times K,$$

Where ζ represents the zeta potential, *dU*/*dP* is the slope of streaming potential versus pressure, and η, ε, ε0 and C represent electrolyte viscosity, dielectric constant, vacuum permittivity and conductivity, respectively.

### Release of SR

All SR-treated samples were soaked in 10-ml phosphate-buffered saline for 1 day, 3 days, 5 days, 7 days, 14 days and 28 days. At each time point, the SR release content was detected using an ultraviolet–visible spectrophotometer (UV–vis, Lambada 750, Perkin Elmer, USA) at 320 nm.

### Cell proliferation

Briefly, 2 × 10^4^ MC3T3-E1 cells were seeded on the surfaces of samples, which were placed in 24-well plates. Three replicates of each group were tested by alamarBlue™ assay (Thermo Fisher Scientific, Inc.) at a predetermined day (1 day, 4 days and 7 days). Culture medium was removed at each time point; residual medium was washed away with 1 ml physiological saline solution (PBS). Then, 0.5 ml medium with 10% alamarBlue™ was transferred to each well. After incubation for 4 h, alamarBlue-specific fluorescence was determined on a microplate reader (Thermo Fisher Scientific, Inc., USA) according to the manufacturer’s instructions [31].

### Cell morphology observation

After culturing on the sample surfaces for 1 day, 4 days and 7 days, MC3T3-E1 cells were rinsed with PBS and fixed with 2.5% glutaraldehyde solution at 4°C. Then, the cells were subjected to step dehydration in graded ethanol solutions (30, 50, 75, 90, 95 and 100 v/v %), and hexamethyldisilane was used as a desiccant. Finally, the cells were sputter-coated with platinum prior to SEM observation [32].

### Alkaline phosphatase staining

Alkaline phosphatase staining was performed on 2 × 10^4^ MC3T3-E1 cells cultured on the sample surface after 7 and 14 days. BCIP/NBT Alkaline Phosphatase Color Development Kit (Beyotime, Shanghai, China) was utilized to check the activity of ALP. Images were captured to show the activity of ALP according to the manufacturer’s instructions.

### Alizarin red staining

Alizarin red staining was performed for 2 × 10^4^ MC3T3-E1 cells cultured on sample surfaces after 7 and 14 days. Alizarin Red S solution (1%, KeyGENBioTECH, Nanjing, China) was incubated with cells fixed using 75% of alcohol solution. Ten minutes later, the cells were washed with ddH_2_O until no dye color remained in solution. Images were captured to show ECM mineralization. Then, the stained samples were eluted in 2% cetylpyridinium chloride (Aladdin, Shanghai, China). The values of optical density (OD) were measured at 620-nm absorbance [33].

### Collagen staining

MC3T3-E1 cells were rinsed with PBS and fixed with 4% paraformaldehyde after culturing on sample surfaces for 7 and 14 days. Alizarin Red S solution was incubated with cells for at least 18 h. Then, samples were flushed with 0.1M acetic acid until no dye color remained in solution. Images were captured to show the secretion of collagen. For quantitative analysis, the stained samples were eluted with NaOH (0.2M) and methyl alcohol solution (1:1), and OD values were measured at 540-nm absorbance [33].

## Results

### Surface characterization

**Figure 1.**
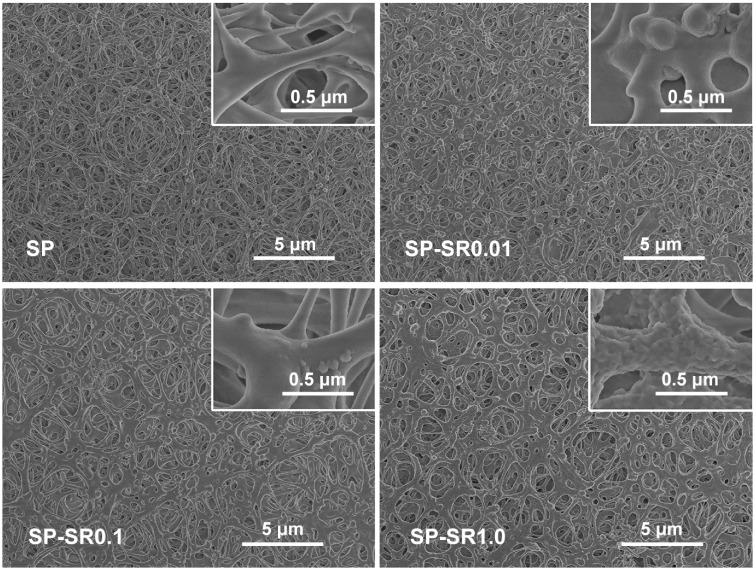
SEM Morphologies of SP (**a**); SP-SR0.01 (**b**); SP-SR0.1 (**c**); SP-SR1.0 (**d**) at low and high magnifications

The surface morphologies of the samples before and after loading SR are shown in Fig. 1. The SP surface was a 3D porous structure. After immersion in SR water solution, drug particles were evenly absorbed onto the special structure. The 3D surface of SR-treated samples was rougher than those of SP only. On the surface of SP-SR0.01, there were sparsely distributed bumps. On the surface of SP-SR0.1, the bump density was increased. On the surface of SP-SR1.0, the surface was covered with continuous folds. These results showed different surface roughness in different solution concentrations.

**Figure 2.**
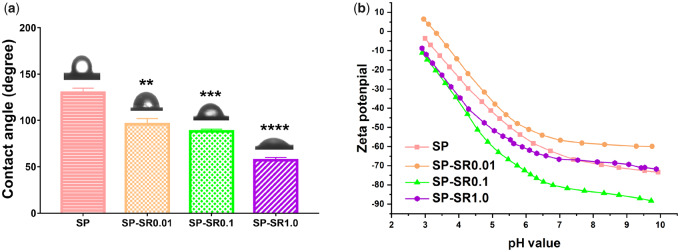
Surface characterization of contact angle (**a**) (**P < 0.01; ***P < 0.001; ****P < 0.0001. All the data are expressed as the means ± SD and *n* = 3); zeta-potential variation versus pH of the potassium chloride solution acquired from the samples (**b**)

The water-contact angle measurements of the samples are shown in Fig. 2a. After loading with SR, the contact angle of the samples significantly decreased compared with that of SP only, which indicated sample hydrophilicity increased with SR concentration. Zeta potentials of the samples are shown in Fig. 2b. Zeta potentials of SP-SR0.1 and SP-SR1.0 were lower than that of SP, but that of SP-SR0.01 was higher than that of SP.

**Figure 3.**
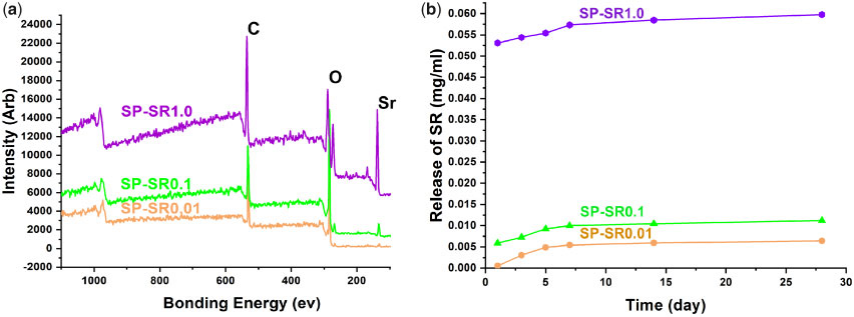
XPS Full spectra of the samples (**a**); release of SR for 1, 3, 5, 7, 14 and 28 days (**b**)

X-ray photoelectron spectroscopy was applied to determine the Sr of the samples, and the results are shown in Fig. 3a. C (at binding energy 535 eV), O (at binding energy 289 eV) and Sr (at binding energy 138 eV) appeared on all samples, and Sr content increased from SP-SR0.01 to SP-SR0.1 and to SP-SR1.0. Table 1 lists the detailed chemical compositions of the samples. The Sr contents of SP-SR0.01, SP-SR0.1 and SP-SR1.0 were 0.08%, 0.23% and 8.97%, respectively. Figure 3b shows the SR release from the samples. In the first week, SR had a stable release. SR release from SP-SR1.0 was highest among all samples. The trend of SR release from the samples was as follows: SP-SR0.01 < SP-SR0.1 < SP-SR1.0.

**Table 1.** Chemical composition of the samples

Sample	C (at%)	O (at%)	Sr (at%)
SP-SR0.01	82.83	16.83	0.08
SP-SR0.1	84.01	15.72	0.23
SP-SR1.0	52.92	35.99	8.97

## Responses of MC3T3-E1 cells

### The adhesion of MC3T3-E1 was strengthened by SP-SR

**Figure 4.**
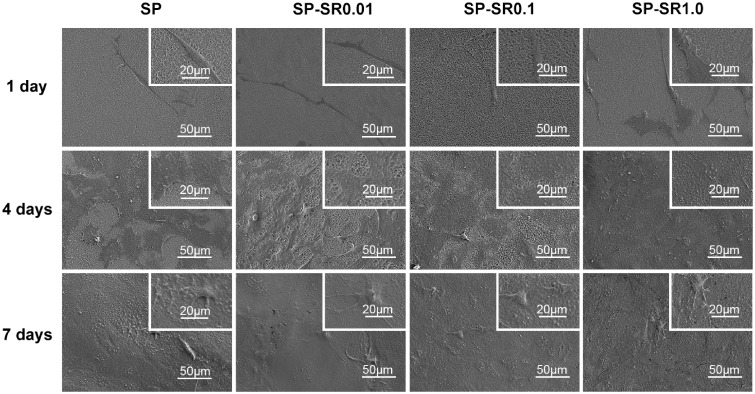
SEM Morphologies of the MC3T3-E1 cell cultured on various sample surfaces for 1 day, 4 days and 7 days at low and high magnifications

To detect the adhesion of MC3T3-E1 cells, we cultured cells on sample surfaces and obtained images at the 1st, 4th and 7th days. Figure 4 shows cell morphology. On the first day, cells maintained a 3D and elongated shape on the surfaces of sulfonated PEEK. As the drug load increased, the cells gradually flattened on the sample surface. After culturing for 4 days, cells were flattened on the sample surface, and the surface of SP-SR1.0 was completely covered by cells. Similarly, cells from all groups eventually covered their respective surfaces after 7 days of culture. Hence, surface-loaded drugs can further promote the adhesion of cells.

### The activity of MC3T3-E1 cells was increased by SP-SR

**Figure 5.**
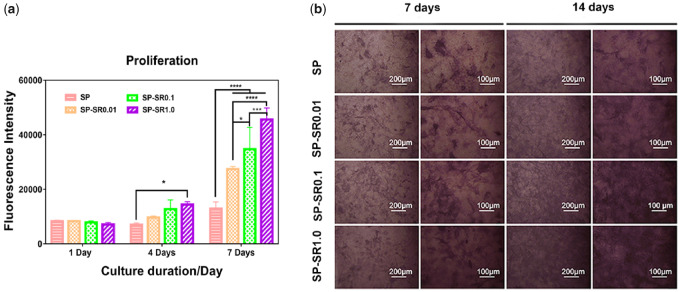
Proliferation and viabilities of MC3T3-E1 cell cultured on the sample surfaces (**a**) (*P < 0.05, **P < 0.01, ***P < 0.001, ****P < 0.0001; all the data are expressed as the means ± SD and *n* = 3); the activity of ALP of the MC3T3-E1 cell cultured on various sample surfaces for 7 days and 14 days at low and high magnifications (**b**)

In this study, we chose osteogenic MC3T3-E1 cell to evaluate bioactivity of sample. Cell proliferation was detected by alamarBlue assay; the results are shown in Fig. 5a. The number of cells was upregulated with increasing drug concentration and time. After culturing for 1 day, there was no significant difference among all the groups. After incubation for 4 days, the cell activity of groups loaded with drugs was slightly higher than that of the SP group. More noticeable differences were observed on the 7th day. Proper drug levels on sulfonated PEEK surfaces can accelerate MC3T3-E1 cell activity.

Alkaline phosphatase is a marker for the early differentiation of osteoblasts. To detect the activity of ALP, we cultured MC3T3-E1 cells on the surfaces of samples for 7 and 14 days; the results are shown in Fig. 5b. As the SR content increased, MC3T3-E1 cell confluency increased over 7 days. The confluent area at 14 days was higher than that at 7 days, and growth was consistent for 7 days.

### The osteogenesis of MC3T3-E1 cells was accelerated by SP-SR

**Figure 6.**
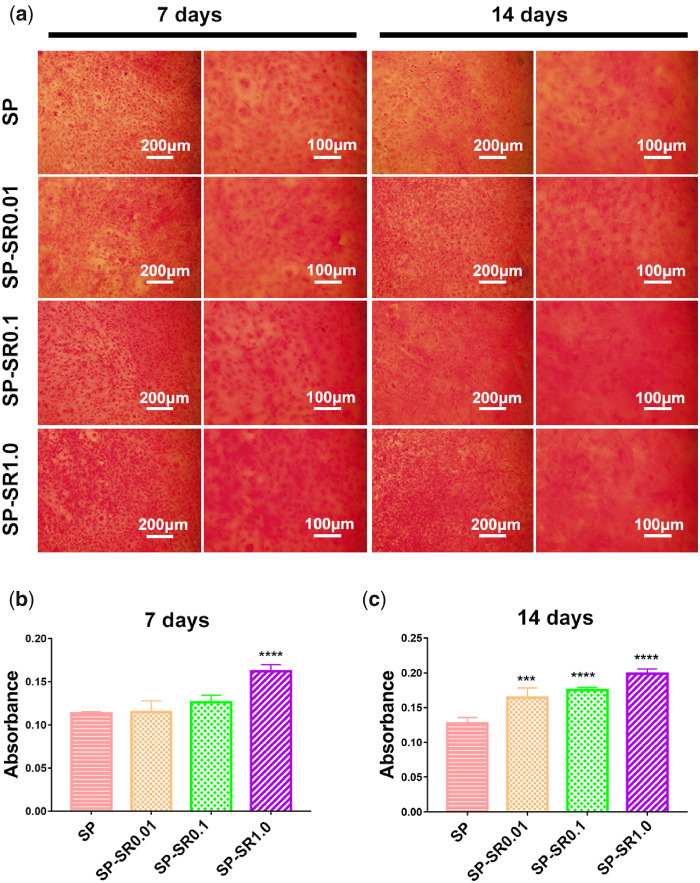
Collagen secretion of the MC3T3-E1 cell cultured on various sample surfaces for 7 and 14 days at low and high magnifications (**a**); quantitative analysis for 7 days (**b**); and 14 days (**c**). (***P < 0.001; ****P < 0.0001. All the data are expressed as the means ± SD and *n* = 3)

Collagen is an important component of bone. To detect MC3T3-E1 osteogenesis, we performed collagen and alizarin red staining by culturing MC3T3-E1 cells on sample surfaces for 7 and 14 days. The results of collagen secretion are shown in Fig. 6. Compared with SP, there were more positive areas on the samples loaded with SR; the positive area increased with SR content at the 7th and 14th days, which indicated that SR facilitated collagen secretion of MC3T3-E1 cells. The quantitative results of cell growth on collagen for 7 and 14 days are shown in Fig. 6b and c. These results were consistent with the stained results. At the 7th day, SP-SR1.0 showed significant differences with SP. At the 14th day, all samples loaded with SR showed significantly higher collagen secretion compared with SP.

**Figure 7.**
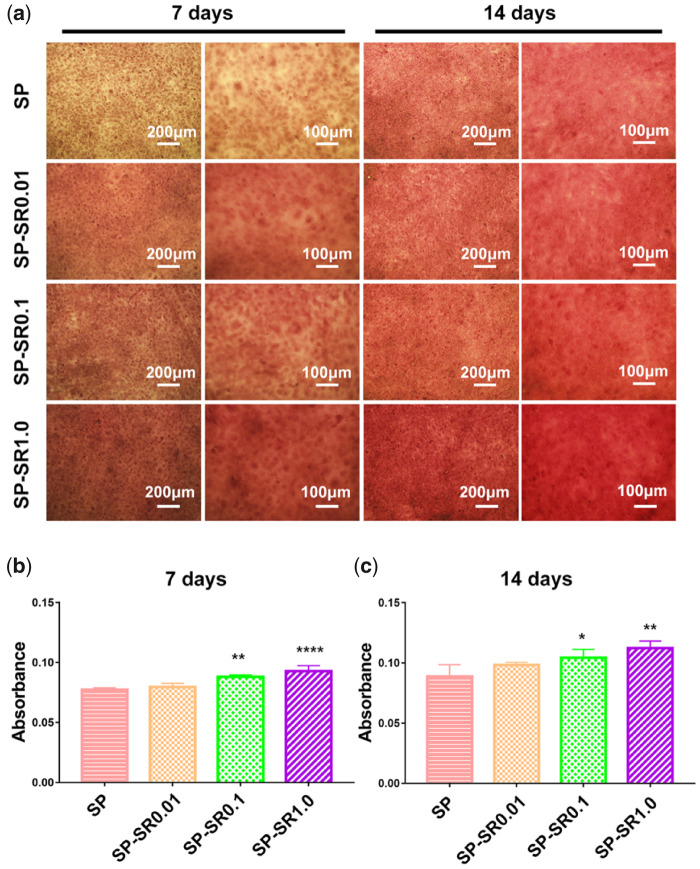
ECM mineralization deposition of the MC3T3-E1 cell cultured on various sample surfaces for 7 and 14 days at low and high magnifications (**a**); quantitative analysis for 7 days (**b**); and 14 days (**c**). (*P < 0.05, **P < 0.01, ****P < 0.0001. All the data are expressed as the means ± SD and *n* = 3)

Figure 7 shows the ECM mineralization deposition of the MC3T3-E1 cells as detected by alizarin red staining. When the cells were cultured on the samples for 7 days, there was more mineralization deposition on samples loaded with SR. The positive area increased with SR content. The ECM mineralization deposition of the cells cultured on the samples for 14 days was higher than that for 7 days. The trend of ECM mineralization deposition of the cells at 14 days was consistent with that for 7 days. The quantitative mineralization deposition of the cells cultured on the samples for 7 and 14 days is shown in Fig. 7b and c. The differences increased with SR content when compared with SP. When the culture time was extended to 14 days, the significance was smaller than that for 7 days.

## Discussion

Polyetheretherketone is FDA-authorized implantation material that has been broadly used in clinic [34]. Polyetheretherketone is a good material choice for prosthetic replacements owing to its several superior properties [35]. Our previous studies showed that sulfonation can form 3D porous layer on PEEK surface, which provides abundant space for drug loading [14]. In adition, the sulfonic acid groups formed by sulfonation could provide chemical combination sites for drug groups. Doxorubicin, chitosan, peptides, pravastatin and short chain fatty acids can be loaded onto the SP surface and delivered for a period of time [15, 30, 33]. In this study, SR was loaded onto SP surfaces (confirmed by XPS). Strontium is an important trace element in humans. Therefore, the strontium loading was beneficial for the bone remodel of OP. Additionally, localized delivery can minimize systemic side effects.

Zeta potential is an index that detects the stability of dispersion systems, that is the higher the absolute value of the zeta potential, the more stable the surface charge [36]. The zeta potential of each group of materials within physiological pH ranged from −50 to −80, indicating that the surface charge of each group of materials was relatively stable [37]. Dynamic cell–material interaction is a complex process, and the surface properties of biological materials can affect the interaction between cells and materials. The charge on the surface of the material is a key factor that regulates cell response especially in the early stage of cell adhesion on the material surface [38]. Additionally, negative zeta potentials on the material surface can promote cell adhesion and proliferation [39]. In this study, SP surfaces loaded with SR molecules decreased the surface zeta potential, indicating that the materials in each group were conducive to cell adhesion and proliferation at physiological pH.

Material surface hydrophilicity is a factor that affects biocompatibility [40]. Studies have shown that hydrophilic titanium surfaces reduce inflammation, which affect the therapeutic effect of implant materials [41]. In addition, the hydrophilicity of titanium surface by means of heat induction helped improve the biological activity of materials [42]. In this study, SR increased the hydrophilicity of SP and improved SP biocompatibility.

The proliferation of MC3T3-E1 cells on the surface was observed on the 1st, 4th and 7th days. There were no significant differences in cell proliferation among the groups on the first day, which may be owing to early incomplete adhesion. After the cells completely adhered to the surfaces of the material on the 4th day, electron microscopy indicated that SP-SR can promote cell proliferation and increase the number of cells.

Strontium ranelate-treated sulfonated PEEK promoted cell adhesion dependent on SR concentration, which enhanced the zeta potential, sample surface roughness and hydrophilicity. Zeta potential is a factor affecting cell adhesion [37]. The cells of the SP-SR0.01 group were thinner and longer than the other groups on the first day, which may be owing to the higher zeta potential on the SP-SR 0.01 surface. The roughness and hydrophilicity of the material surface also affect adhesion [43–45]. Cells on the surface of SP-SR1.0, which was the roughest and most hydrophilic, were well-stretched, indicating that SP-SR was beneficial to cell adhesion. After 4 days, there were no significant differences in the morphology of the cells among the group; this may have been because of the reason that the material surfaces were mostly confluent, resulting in consistent material surface properties.

Chemical elements, such as zinc [1], magnesium [46], calcium [47] and strontium [48], have been shown to exhibit superior properties for bone regeneration by promoting bone-related cell proliferation, differentiation and osteogenesis-related genes expression [49–51]. Recently, SR has received more attention owing to its ability to simultaneously modulate osteoclasts and osteoblasts through several pathways [28, 52]. SR can antagonize the activation of nuclear factor kappa-light-chain-enhancer of the activated B cells (NF-κB) and thus affects the osteoclastogenesis via the OPG\RANKL\RANK pathway [21, 53]. Also, SR can promote osteogenic differentiation of rat bone mesenchymal stem cells via the BMP-2/Smad pathway [54, 55] and improve osteoblast function by activating FGF receptors [56]. Alkaline phosphatase is a marker for the early differentiation of osteoblasts. Collagen and inorganic matter are both important components of bone [57]. In this study, we found that SP-SR enhanced the activity of ALP, elevated collagen secretion and promoted the mineralization of the extracellular matrix of MC3T3-E1 at the differentiation and maturation stage, that is SP-SR plays a beneficial role on bone formation. Similar to our findings, Fernandez *et al.* [58] and Wang *et al.* [59] reported that SR can regulate bone matrix mineralization by promoting the activity of ALP .

## Conclusion

In this study, SR was loaded onto 3D porous PEEK surface. Strontium ranelate can stably release within first 7 days, which is important for early-stage bone cell proliferation and differentiation. After loading with SR, the contact angle of the samples significantly decreases with increasing SR content, the zeta potential of the samples loaded with SR is lower than that of sulfonated PEEK. We studied the cell response of samples with different contents of SR. The results showed that SR loading samples can enhance cell proliferation, the activity of ALP, collagen secretion and ECM mineralization deposition compared with SP. Collectively, SP loading with SR can be an alternative way for OP patients in clinic.

## Funding

This study was supported by grants from the National Natural Science Foundation of China (81403029), the Natural Science Foundation of Shanghai (19ZR1449100), the Science and Technology Commission of Shanghai Municipality (19JC1415500), the Science and Technology Commission of Shanghai Municipality (18410760600), and the International Partnership Program of Chinese Academy of Sciences (Grant No. GJHZ1850). 

*Conflict of interest statement*. All authors declare there are no coflicts of interest. 
